# Enhancing infection control activities through departmental infection control facilitators

**DOI:** 10.1017/ash.2025.100

**Published:** 2025-09-03

**Authors:** Sunhwa Kim

**Affiliations:** Affiliation including department and/or program: Infection control department of Gangnam severance hospital

**Keywords:** Healthcare associated infection, Quality improvement, Collaboration, Intensive care unit, central venous catheter

## Abstract

**Introduction:** In the Intensive Care Unit(ICU), healthcare-associated infections can arise from factors such as compromised patient immunity and the use of diverse medical equipment. Furthermore, inadequate awareness of infection control among ICU staff can further increase the risk of infections. Therefore, it is crucial for ICU staff to recognize and address infection risks proactively. To enhance infection control measures, designated infection control facilitators within the department have spearheaded infection control activities. **Case Presentation:** Internal assessments within the ICU identified areas requiring improvement in infection control, leading to the formulation of a self-improvement initiative. The evaluation results revealed deficiencies in pre- hub disinfection and the appropriateness of Chlorhexidine gluconate(CHG) bathing. To address this, ICU team members were tasked with monitoring hand hygiene and performing pre-hub disinfection at least 10 times before central venous catheter usage. The monitoring results were shared with department members monthly, encouraging performance improvement by rewarding outstanding employees. Additionally, protocols and educational videos for proper CHG bathing were developed within the ICU and reviewed by the Infection Control Department. Using this material, internal education sessions were conducted within the ICU to support all team members in achieving their goals. **Discussion:** Through various improvement initiatives, staff awareness of infection control has increased, leading to proper CHG bathing and hub disinfection. The incidence rate of central venous catheter-related bloodstream infections decreased from 4.25 in 2022 to 3.35 in 2023. Additionally, hand hygiene compliance increased from 92% in 2022 to 96% in 2023. For effective infection management, the participation of not only the Infection Control Department but also departmental members is crucial. Through effective collaboration and discussions between ICU staff and the infection control team, we were able to address departmental issues, improve staff awareness and performance in infection management. Sustained interest and participation in these activities require continuous staffing and support.

